# HIV-1 Superinfection in Women Broadens and Strengthens the Neutralizing Antibody Response

**DOI:** 10.1371/journal.ppat.1002611

**Published:** 2012-03-29

**Authors:** Valerie Cortez, Katherine Odem-Davis, R. Scott McClelland, Walter Jaoko, Julie Overbaugh

**Affiliations:** 1 Molecular and Cellular Biology Graduate Program, University of Washington, Seattle, Washington, United States of America; 2 Department of Epidemiology, University of Washington, Seattle, Washington, United States of America; 3 Division of Human Biology, Fred Hutchinson Cancer Research Center, Seattle, Washington, United States of America; 4 Department of Medical Microbiology, University of Nairobi, Nairobi, Kenya; University of Zurich, Switzerland

## Abstract

Identifying naturally-occurring neutralizing antibodies (NAb) that are cross-reactive against all global subtypes of HIV-1 is an important step toward the development of a vaccine. Establishing the host and viral determinants for eliciting such broadly NAbs is also critical for immunogen design. NAb breadth has previously been shown to be positively associated with viral diversity. Therefore, we hypothesized that superinfected individuals develop a broad NAb response as a result of increased antigenic stimulation by two distinct viruses. To test this hypothesis, plasma samples from 12 superinfected women each assigned to three singly infected women were tested against a panel of eight viruses representing four different HIV-1 subtypes at matched time points post-superinfection (∼5 years post-initial infection). Here we show superinfected individuals develop significantly broader NAb responses post-superinfection when compared to singly infected individuals (RR = 1.68, CI: 1.23–2.30, p = 0.001). This was true even after controlling for NAb breadth developed prior to superinfection, contemporaneous CD4+ T cell count and viral load. Similarly, both unadjusted and adjusted analyses showed significantly greater potency in superinfected cases compared to controls. Notably, two superinfected individuals were able to neutralize variants from four different subtypes at plasma dilutions >1∶300, suggesting that their NAbs exhibit elite activity. Cross-subtype breadth was detected within a year of superinfection in both of these individuals, which was within 1.5 years of their initial infection. These data suggest that sequential infections lead to augmentation of the NAb response, a process that may provide insight into potential mechanisms that contribute to the development of antibody breadth. Therefore, a successful vaccination strategy that mimics superinfection may lead to the development of broad NAbs in immunized individuals.

## Author Summary

A broad and potent antibody response is considered essential for an effective HIV-1 vaccine that will protect against diverse circulating strains. Consequently, there is great interest in both the host and viral factors that impact the development of the neutralizing antibody (NAb) response in natural HIV-1 infections. HIV-infected individuals who become superinfected with a second virus from a different source partner represent unique cases for studying the antibody response, as superinfection reflects exposure to different HIV-1 antigenic variants, and hence may provide insight into the development of broadly NAbs. In support of this model, we show here that superinfected individuals develop broader and more potent NAb responses than singly infected individuals, a result that is likely due to the increased antigenic stimulation from two viruses compared to one. Our findings remained unchanged after controlling for other factors that have been shown to influence the NAbs response, such as CD4+ T cell count and viral load. This study demonstrates that superinfection yields antibodies that have the capacity to recognize diverse circulating HIV-1 variants. Therefore, further characterization of these superinfected individuals' NAb responses could lead to novel insights into pathways that elicit broadly NAbs.

## Introduction

Multiple studies have demonstrated the potential of HIV-specific neutralizing antibodies (NAbs) to protect against infection using nonhuman primate models [1], [2]. However, it remains unclear how to elicit a NAb response of sufficient breadth and potency to protect humans against diverse circulating HIV-1 variants, which can differ by several orders of magnitude in neutralization sensitivity [1], [2]. Therefore, investigating naturally-occurring antibody responses that can neutralize viruses across the major viral subtypes remains a major focus of research [3]. In the past few years, multiple HIV-specific broadly neutralizing monoclonal antibodies have been isolated from HIV-infected individuals with elite neutralizing activity [4]–[8]. This subset of individuals comprises about 1% of chronically-infected individuals and are considered elite neutralizers based on their ability to potently neutralize viruses from multiple subtypes [9]. The collection of broad monoclonal antibodies identified to date, which were isolated more than a decade after initial HIV-1 infection in some cases, have undergone extensive somatic hypermutation, a process that would be difficult to mimic with a HIV-1 vaccine [2], [10]. Also, these monoclonal antibodies have been isolated from individuals who were presumably infected with a single HIV-1 strain, although in most cases, the possibility of superinfection (SI) was not addressed. Within singly infected populations, NAb breadth has been positively associated with viral diversity [11]. Therefore, individuals infected with multiple HIV-1 strains as a result of SI by a second source partner may generate broadly NAbs in response to stimulation from both viruses.

Initially, it was hypothesized that SI resulted from a weak NAb response that was unable to protect the individual from reinfection. A small study of three SI cases and three viral strains provided some support for this model [12]. However, in a larger study using a panel of 16 viruses from a number of different subtypes, Blish et al. showed no significant differences in the NAb breadth or potency in six superinfected cases immediately before acquisition of the second virus compared to 18 singly infected controls at matched time points [13]. In this study, where the focus was on correlates of protection from SI, the NAb repertoire and breadth developed in the years following SI were not examined.

In the past year, two studies have provided evidence of a broadening of the NAb response after SI. In a South African individual that became superinfected 13–15 weeks post-initial infection, broad and potent responses were detected 3 years post-initial infection (32 of 42 heterologous viruses neutralized, some at plasma dilutions >1∶10,000) [14]. However, it was not possible to determine whether SI is typically associated with a broad NAb response or if this single case was merely coincidental. Powell et al. aimed to address this question by measuring the difference in NAb responses from four superinfected cases and 23 singly infected controls against seven regional primary isolates from Cameroon and two subtype B viruses [15]. They showed that the average change in NAb breadth and potency between pre-SI and post-SI evaluations for superinfected cases was significantly greater than that of non-superinfected controls [15]. While this analysis provides some evidence that superinfected individuals develop broader NAb responses than singly infected individuals, the results have to be interpreted in the context of a number of limitations in the cohort studied, including an imbalanced distribution of cases and controls where matching was based on two groups of controls rather than an equal ratio of individuals. More significantly, since seroconversion dates were unknown for many individuals, the control groups were assigned based on the time each case had participated in the study, rather than how long they had been infected. Therefore, time since infection, which has been strongly associated with the development of NAb breadth [16]–[18], could not be accounted for in this analysis. Furthermore, due to the small number of superinfected cases, the investigators were unable to control for potential confounding factors such as CD4^+^ T cell count and viral load, which also impact NAb breadth [11], [16]–[19]. Nonetheless, these cases are intriguing and highlight a need for a controlled study with greater numbers of superinfected cases with appropriately matched controls.

We have previously identified 12 superinfected individuals from a cohort of high-risk women in Mombasa, Kenya by comparing partial *env* and *gag* sequences over a 5-year period beginning with initial infection [20]–[22]. This cohort includes individuals initially infected by viruses from multiple subtypes (A, C, and D), and subsequently reinfected by intra or intersubtype viruses. Here we describe the results from our nested cohort study in which we tested the hypothesis that superinfected individuals develop broader and more potent NAb responses compared to non-superinfected individuals as a result of increased antigenic stimulation by two distinct viruses. Our findings illustrate that SI leads to an augmentation of the NAb response and thus, provides significant support for SI as a useful model for studying the development of the NAb response to diverse HIV-1 antigens.

## Results

### Cohort characteristics

**Figure 1:**
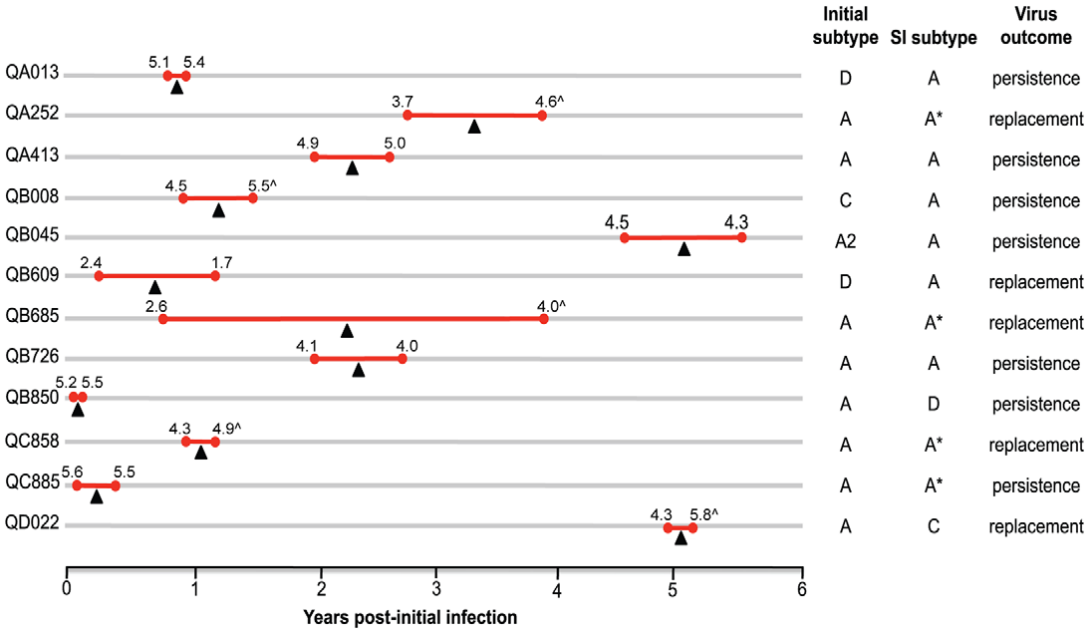
Viral characteristics of 12 cases of SI. Subject IDs are shown to the left with time since initial infection plotted at the bottom. Red bars denote the interval during which SI occurred, with closed circles representing the last time point in which SI was last undetected and the time point at which SI was first detected. Black triangles mark the midpoint of this interval, which was used as the estimated time of the SI event. The individual's viral load (log_10_ copies/ml) at the corresponding time points is noted above each red bar, with (∧) denoting increases in viral load >0.5 log_10_ copies/ml. Subtyping in the two columns on the right are based on *env* sequences. However, the data for those individuals with an (*) by their superinfecting virus subtype based on *gag* sequences alone as the superinfecting *env* sequence was not detected at time points tested. Virus outcome was based on the longitudinal analyses previously described [20]–[22]. Persistence was defined as the detection of co-existence of the superinfecting virus with the initial virus following its introduction, while we classified replacement superinfections as the dominance of the superinfecting virus based on lack of detection of the initial virus post-SI [22].

The 12 cases of SI demonstrated considerable heterogeneity with respect to the temporal occurrence and virologic factors related to their superinfections (Figure 1) [20]–[22]. Some women were superinfected soon after initial infection, while others became superinfected much later during chronic infection (range: ∼2 months to 5 years post-initial infection), with the median occurrence at 1.72 years post-initial infection. Eight individuals (60%) experienced an increase in viral load after SI; in three cases the increase was very small (<0.5 log_10_ copies/ml), while in the remaining five the mean change was 1.08 log_10_ copies/ml. Similar numbers of women had inter and intrasubtype superinfections based on *env* and *gag* sequences, and all women were infected with at least one subtype A virus, the dominant subtype in Kenya [23]. Based on the longitudinal analyses previously described [20]–[22], the superinfecting strain persisted in combination with the initial virus in seven (58%) of the women, whereas it appeared to have largely replaced the initial virus in the other five (42%) (Figure 1).

Three non-superinfected individuals, selected from a pool of women identified as singly infected in prior screens [20]–[22], were matched to each superinfected case by the initial infecting virus subtype, time post-initial infection, and sample availability. NAb breadth and potency were analyzed both pre and post-SI. The pre-SI time point for each superinfected case and her matched controls varied in relation to initial infection depending on the timing of the SI event. The post-SI time point was evaluated at a single time point for all individuals an average of 5 years post-initial infection, when all 12 cases had been superinfected for at least 1 year. The single post-SI time point enabled us to draw comparisons across the entire cohort after the development of the NAb response to both the initial and superinfecting viruses, yet before the onset of overt immunodeficiency [16], [24]–[26]. Superinfected women had significantly lower mean viral loads than singly infected women pre-SI (Log_10_VL: 4.24 vs. 4.79, respectively; p = 0.034), but were comparable post-SI (Log_10_VL: 4.78 vs. 4.89, respectively; p = 0.699). Both groups also had similar mean CD4+ T cell counts post-SI (370 vs. 380; p = 0.886). Insufficient CD4+ T cell count data were available for the cohort at the time points prior to SI for analysis. Superinfected women had unprotected sex on average 22% of the time compared to 33% of the time for non-superinfected women (p = 0.175) and both groups had similar numbers of partners per week (0.66 vs. 0.49, respectively; p = 0.069). All individuals in this study were ARV naïve at the time points examined.

### Comparison of NAb breadth and potency in superinfected and singly infected women after SI

Eight viruses from subtypes A, B, C, and D that exhibit varying degrees of neutralization sensitivity to monoclonal antibodies as well as pooled plasma derived from HIV-1 infected individuals in Kenya were used to measure NAb breadth and potency both pre and post-SI (Table 1). Seven of the eight viruses were isolated from individuals during acute infection, and the majority were Tier 2 variants [27].

**Table 1:** Neutralization sensitivities of eight HIV-1 envelope variants of different subtypes.

Pseudovirus	Subject/transmission route	Time postinfection^a^ (days)	Subtype	Tier^b^	IC_50_ for pooled plasma^c^	IC_50_ (ug/ml) for indicated monoclonal					Source or reference
						VRC01	PG9	b12	2F5	4E10	
**Q461.d1**	female/heterosexual	17	A	1B	489	0.29	1	1	0.18	0.11	43
**Q769.b9**	female/heterosexual	56	A	2	52	0.17	0.05	>20	20	>20	43
**Q259.d2.26**	female/heterosexual	17	A	2	59	0.55	>1	>20	>20	>20	43
**Q842.d16**	female/heterosexual	49	A	2	136	0.51	0.08	>20	>20	14.6	43
**QD435.100M.a4**	female/heterosexual	100	D	2	69	0.36	>10	5.74	0.76	2.58	43
**QC406.70M.f3**	female/heterosexual	70	C	2	119	>1	0.15	>20	>20	>20	44
**DU156.12**	female/heterosexual	28	C	2	195	0.31	0.16	3.13	>20	1.57	46
**SF162**	male/MSM^d^	Chronic	B	1A	583	0.6	>1	0.01	4.5	8	45

a This estimate is based on published data and methods for estimating time of infection as described [54].

b Tier designation is based on available data [27] or, in some cases, on extrapolation using the IC50 values shown here.

c Pooled plasma was from 30 HIV-positive Kenyan individuals from the cohort.

d MSM, men who have sex with men.

**Figure 2:**
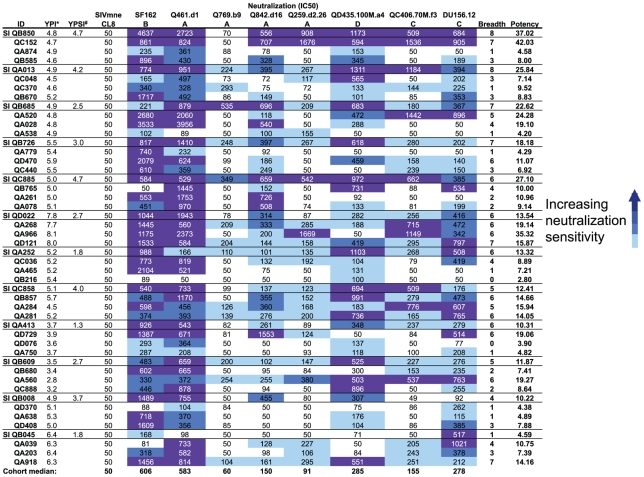
Post-SI neutralization profiles for superinfected and non-superinfected plasmas tested against heterologous HIV-1 variants. Subject IDs are shown to in the first column. Superinfected individuals denoted with borders and “SI” before ID number, with the three matched controls listed below. The next columns lists the years post initial infection (YPI*) that was tested, which was used to match cases and controls. The following columns list the years post-SI (YPSI^#^) for each superinfected case. All samples were chosen near 5 years post-initial infection. However, some samples were taken 1–2 years before or after this time point because of sample availability. Subsequent columns contain the IC50 for each plasma-virus pair, which is the reciprocal dilution of plasma that led to a 50% reduction in infectivity. Plasma samples that showed neutralization below the limit of detection were designated an IC50 value of 50, the midpoint between our starting dilution (1∶100) and 0. IC50s are shown as a heat map to represent increasing neutralization sensitivity, with white boxes for values below 100, light blue boxes for values between 101 and 300, darker blue boxes for values between 301 and 500, and the darkest blue boxes for values greater than 501. The NAb response breadth and potency scores that are shown here were calculated after taking the average log_2_ IC50s from the two experiments.

**Figure 3:**
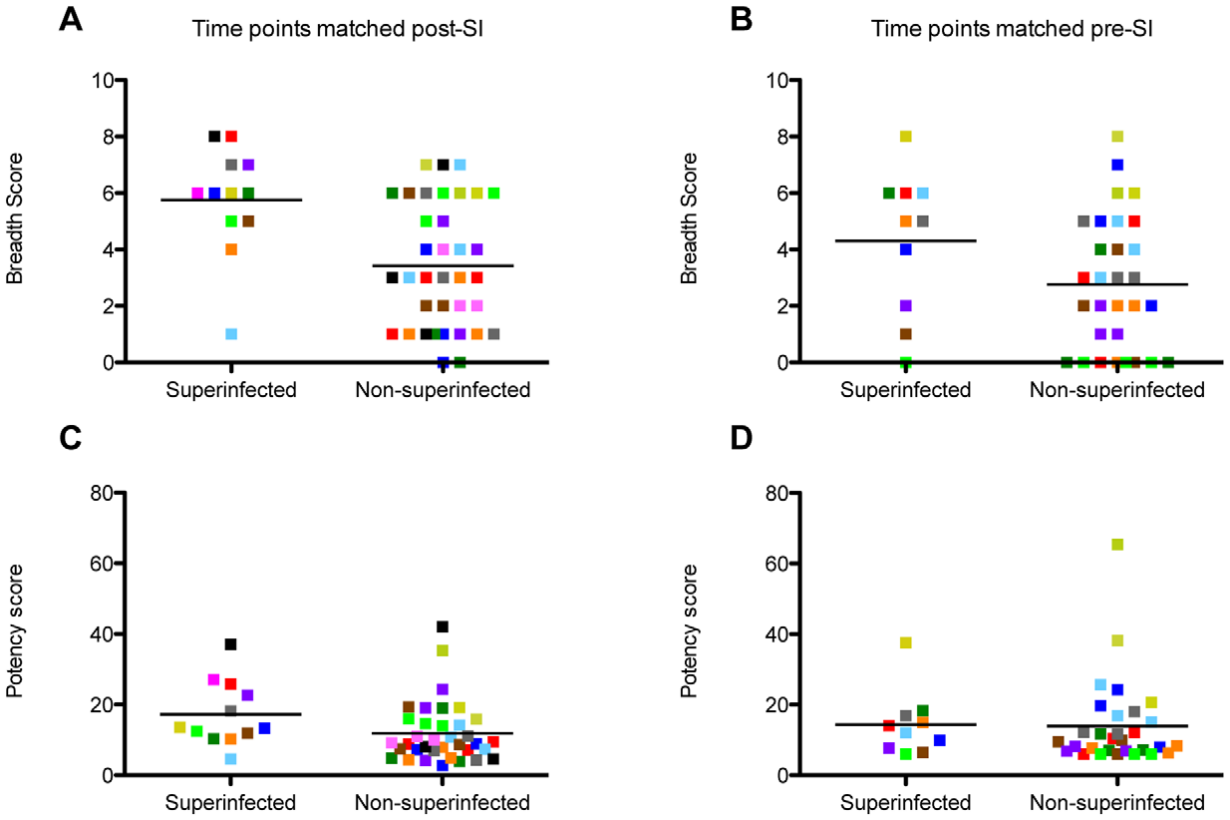
Summary of differences in NAb breadth and potency scores between superinfected and non-superinfected women. Each case and the three matched controls are denoted by a single color. Mean scores shown with horizontal bar. Breadth and potency comparisons post-SI are shown in panels (**A**) and (**C**), respectively. Breadth and potency comparisons pre-SI are shown in panels (**B**) and (**D**), respectively.

To evaluate whether harboring two viruses compared to a single virus influences the development of NAb breadth and potency, we first tested all superinfected cases and matched controls at a time point post-SI, approximately ∼5 years post-initial infection (median time after initial infection: 5.01 years, Range: 2.8–8.1 years) (Figure 2). Overall, the Tier 1 viruses that were neutralization sensitive (SF162 and Q461d1) had the highest median IC50 values (606 and 583, respectively) for the cohort as a whole, while the median IC50s for the six Tier 2 viruses were all below 300. Geometric mean IC50s averaged across the entire panel were significantly different between superinfected cases and singly infected individuals (326.19 vs. 193.33, respectively; p = 0.038). Furthermore, differences in neutralization potency between individual superinfected cases and matched controls are evident, most notably with the more neutralization resistant viruses such as Q769.b9 and Q259.d2.26 (Figure 2). Breadth and potency scores were calculated by normalizing the IC50 of each plasma-virus pair to the cohort median IC50, as described [13]. The mean NAb breadth score of the 12 superinfected women was 5.75, while the mean breadth score of the 36 non-superinfected women was 3.42 (Figure 3A). Superinfected women had, on average, 1.68 (CI: 1.23–2.30, p = 0.001) times greater breath than non-superinfected women (Table 2). Similarly, the mean potency score in superinfected women was higher than singly infected women (17.25 vs. 11.84, respectively) (Figure 3B), and superinfected women had 1.46 (CI: 1.03–2.06, p = 0.033) times greater potency than the non-superinfected group (Table 2).

**Table 2:** Association between NAb breadth and superinfection.

	Breadth			Potency		
Univariate	RR	95% CI	P value	Ratio	95% CI	P value
	1.68	1.27, 2.30	0.001	1.46	1.03, 2.06	0.033

Multivariate adjusting for	aRR	95% CI	P value	aRatio	95% CI	P value
Breadth/potency pre-SI	1.56	1.11, 2.18	0.009	1.40	1.05, 1.86	0.020
Contemporaneous viral load	1.68	1.22, 2.31	0.001	1.54	1.07, 2.25	0.022
Contemporaneous CD4+ T cell count	1.70	1.28, 2.25	<0.001	1.63	1.10, 2.43	0.016
All of the above	1.51	1.01, 2.25	0.040	1.68	1.28, 2.20	<0.001

### Comparison of NAb breadth and potency in superinfected and singly infected women before SI

**Figure 4:**
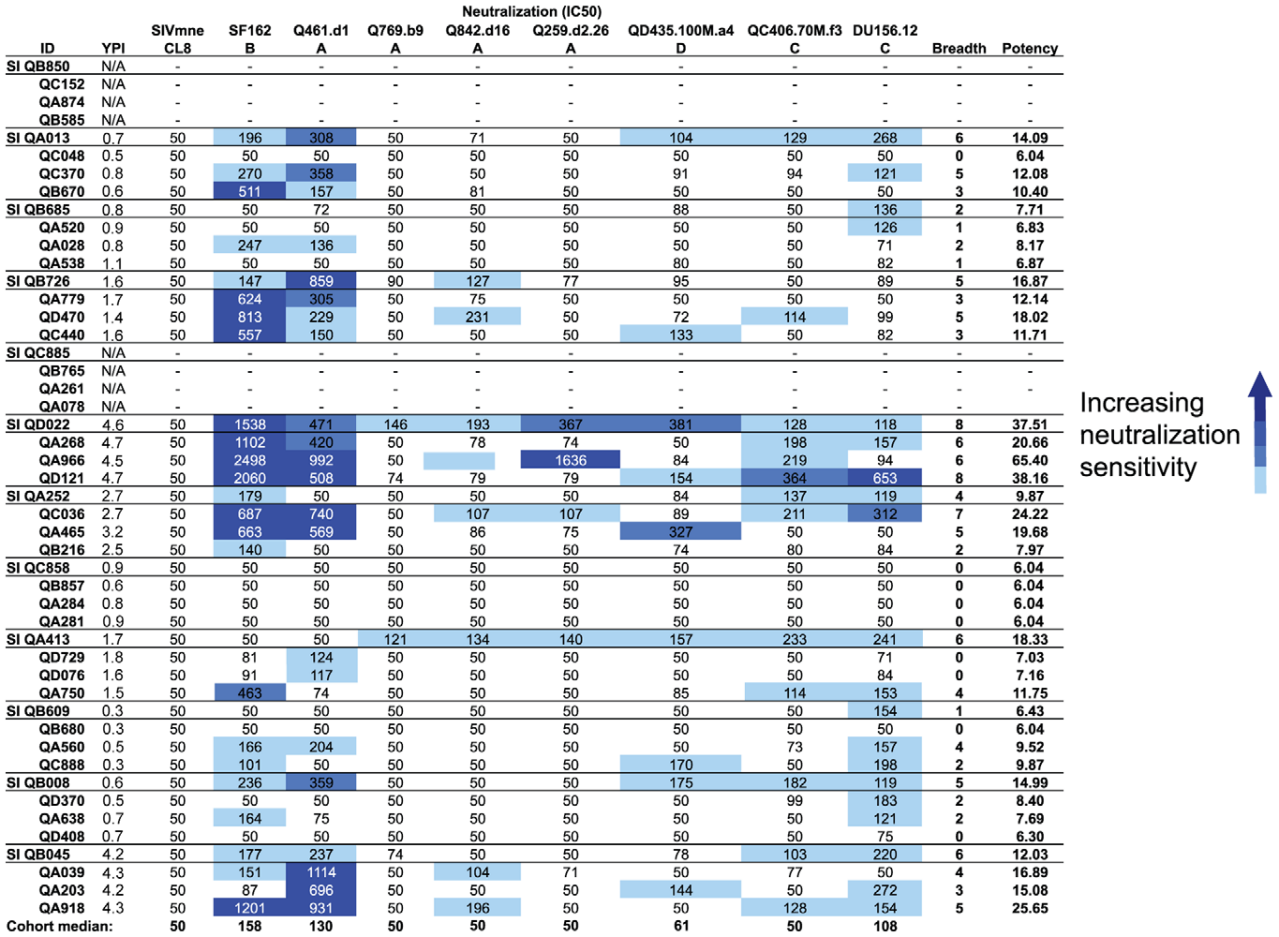
Pre-SI neutralization profiles for superinfected and non-superinfected plasmas tested against heterologous HIV-1 variants. The layout for this figure is as described in the legend for Figure 2. Heterologous HIV-1 variants tested were the same as in the post-SI screen. IC50s are similarly shown with darker colors denoting greater neutralization sensitivity. Plasmas not tested are indicated by a pair of dashes.

In order to determine whether the greater NAb breadth exhibited by superinfected women was independent of any effect of the NAb breadth developed prior to the SI event, we assessed the NAb responses elicited by the initial virus prior to SI in each individual (Figure 4). Viruses that were neutralization sensitive (SF162 and Q461d1) had the highest median IC50 values (158 and 130, respectively), followed by two Tier 2 viruses, DU156.12 and QD435.100M.a4 (108 and 61, respectively). The cohort median IC50s was 50 for the other four Tier 2 viruses, with only a few individuals able to neutralize these viruses at greater than 50% at the lowest dilution tested. Overall, the geometric mean IC50s averaged across the entire panel were not significantly different between superinfected cases and singly infected controls (98.16 vs. 86.02, respectively; p = 0.378). The mean NAb breadth score for superinfected women was 4.30, but only 2.80 for non-superinfected women (Figure 3C). Superinfected women had, on average, 1.50 (CI: 1.05–2.14, p = 0.03) times greater breath than non-superinfected women at the matched pre-SI time points. However, upon adjusting for contemporaneous viral load, which differed between the two groups and is a correlate of NAb breadth [16], [19], the difference in breadth at this time point was attenuated and no longer statistically significant (RR = 1.45, CI: 0.97–2.16, p = 0.067). We observed comparable mean potency scores between superinfected and singly infected women (14.39 vs. 13.93, respectively) (Figure 3D), that were not significantly different in our univariate analysis (p = 0.447), nor after adjusting for contemporaneous viral load (p = 0.195).

### Multivariate modeling of the NAb breadth and potency after SI

We performed a multivariate analysis to examine the relationship between SI status and the NAb response while adjusting for breadth/potency scores pre-SI as well as contemporaneous viral load and CD4^+^ T cell count. We found that none of these factors individually had a major impact on the association between SI and NAb breadth post-SI, as our estimate of 1.68 remained statistically significant with estimates ranging between 1.56 to 1.70 (Table 2). Adjusting simultaneously for all three variables similarly did not substantially change the original estimate (RR = 1.51, CI: 1.01–2.25, p = 0.040) (Table 2). The multivariate analysis for NAb potency post-SI after adjusting for these same three variables yielded a higher estimate, changing from 1.46 to 1.68, illustrating that the association between SI and potency is stronger once these variables are accounted for.

We next performed the same analysis with a modified breadth scoring method previously used by Simek et al. and the Center for HIV-1 AIDS Vaccine Immunology (CHAVI) for Protocol 008 [9]. Using this method, we similarly found that superinfected individuals demonstrated greater breadth than singly infected individuals post-SI (mean scores: 0.75 vs. 0.57, respectively; p = 0.002) (Table S1). Pre-SI breadth also showed differences between superinfected and non-superinfected individuals (mean scores: 0.46 vs. 0.34, respectively; p = 0.019), but this difference did not remain significant after adjusting for contemporaneous viral load (p = 0.435). To determine if our results were sensitive to the exclusion or inclusion of a particular virus in the panel, we performed a stepwise sensitivity analysis where we assessed breadth after removing one virus at a time and also with various combinations of viruses from the original 8-virus panel (e.g. only subtype As, only viruses with a particular neutralization profile, such as resistance to b12 or pooled plasma). Overall, the results were similar to our original finding, with estimates from regression analysis ranging from 1.36 to 1.91, all of which represent statistically significant differences between the two groups (Table S1). In addition, estimates using percent neutralization at a fixed dilution of plasma (1∶100, 1∶200 or 1∶400), as was used in prior studies [9], [15], [28], yielded similar results with statistically significant point estimates ranging from 1.55 to 1.70 (Table S2). Moreover, breadth scores calculated from the percent neutralization at a single dilution were also highly correlated with the breadth scores based on IC50s calculated from full neutralization curves with all six serial dilutions (Spearman's rho range: 0.76–0.88, all with p = <0.0005) (Table S3).

### Longitudinal analysis shows NAb breadth developed early after SI in women with the broadest responses

**Figure 5:**
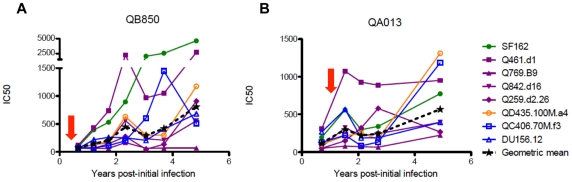
Elite neutralizers QB850 and QA013 develop breadth within 1 year following SI. The kinetics of the NAb responses for QB850 (A) and QA013 (B) are shown versus years post-initial infection. Viruses of the same subtype are shown with the same line color. Geometric mean IC50s for the entire 8-virus panel are shown in the dashed black lines. The approximate time of SI, calculated as described in Figure 1, is indicated by the corresponding red arrows for each individual.

To determine whether SI led to a rapid enhancement of the NAb response, we analyzed longitudinally banked plasma samples from the two women with the broadest responses post-SI (QA013 and QB850) against the same 8-virus panel described above, beginning with the time point available following the initial detection of SI (Figure 5). We found that QA013, who was superinfected ∼11 months after her first infection, experienced a boost in NAb activity immediately following SI (pre-SI geometric mean (GM) IC50 = 118, range: 50–308, ∼0.6 years post-SI GM IC50 = 299, range: 79–1073). Her response continued to increase in potency, with a GM IC50 of 567 at ∼4.2 years post-SI (range: 224–1311). QB850, who was superinfected ∼2 months after her first infection, at first displayed modest cross-subtype activity ∼1 year post-SI (GM IC50 = 146, range: 65–428) before gradually developing elite activity around ∼2.2 years post-SI (GM IC50 = 451, range: 168–2268), which was ∼2.3 years post-initial infection. Overall, these data demonstrate that QB850 and QA013 developed cross-subtype neutralization within 1 year following SI, with two different trajectories that both led to elite NAb activity. To further assess the breadth of the response of these two individuals, additional viruses were tested, with an emphasis on subtypes that were represented by a single virus in our screen (subtypes B and D). There are limited options for subtype D viruses, but we chose a neutralization resistant variant from the Mombasa cohort, QB857. Three subtype B viruses JR-CSF, 6535.3 and CAAN5342.A2, were chosen based on their use in a prior screen to define elite neutralizers [9]. Both QA013 and QB850 plasma samples from 5 years post-initial infection neutralized all four of these viruses, with IC50s ranging from 72–810. The IC50 values we observed for QA013 and QB850 against the subtype B viruses (JR-CSF: 207 and 163, 6523.3: 322 and 810, CAAN5342.A2: 127 and 106, respectively) were all comparable or above the cohort GM IC50s observed in the elite neutralizer screen [9]. However, it was not possible to directly compare all of our IC50s to the top 1% of elite neutralizers identified in that study because the IC50 values for those individuals against these viruses were not presented.

## Discussion

In this study, we tested the ability of antibodies present in superinfected and singly infected women to neutralize a spectrum of circulating HIV-1 variants and thus, discern whether antigenic stimulation by two viruses compared to one has an effect on the subsequent NAb response. Our results demonstrate that the NAb response of superinfected women is significantly broader and more potent than that of singly infected women when compared at matched time points. These data suggest that SI elicits a substantial enhancement of the NAb response with regards to cross-reactivity in the years following reinfection. This conclusion is supported by our analysis controlling for NAb breadth/potency prior to SI and clinical measures that are associated with NAb breadth such as CD4+ T cell count and viral load [11], [16]–[19]. Therefore, the study of superinfected individuals may yield additional insights into the development of broad and potent NAb responses to diverse HIV-1 antigens.

The majority of superinfected individuals exhibited breadth and potency scores that were greater than the average of their matched controls post-SI. Among the SI cases, the three women with the most potent responses (QB850, QA013, QC885) experienced intersubtype superinfections that were characterized by persistence of both the initial and superinfecting viruses. While we attempted to parse out which factors relating to SI may be influencing the generation of a broad response, we were unable to do so conclusively due to a limited sampling of 12 cases (data not shown). However, the characteristics of these three women suggests that continuous stimulation by two distinct viruses from different subtypes may be critical to the induction of broad NAbs. Still, other factors, including the antigenic nature and replication potential of the infecting viruses, may also be important. Conversely, we found a minority of superinfected individuals (QB045, QC858, QD022) exhibited breadth and potency scores that were lower than that of the average from their matched controls. As all three had a CD4+ T cell count >400 post-SI, it is unlikely that lower breadth and potency simply reflected a lack of T cell help that could arise at terminal stages of infection. Some potentially informative features of these cases are that two of the three (QB045 and QD022) are the only individuals in the cohort who became superinfected late after initial infection (>4 years post-initial infection), suggesting that perhaps the timing or duration of either or both infections may be critical to the development of NAb breadth. Moreover, in QD022 and QC858 the superinfecting variant became dominant as the initial virus could no longer be detected post-SI, at least at levels captured by Sanger sequencing, suggesting that continued antigenic stimulation by both infecting viruses may be important in augmenting the NAb response. Additional studies that include deep sequencing of viruses in tissue and plasma would be one starting point to further explore this hypothesis.

In one individual, QA413, multiple Tier 2 viruses were neutralized prior to SI, while the Tier 1 viruses (SF162 and Q461d1) were not. This may suggest that there are unique epitopes common to the two Tier 1 viruses that are not present in the Tier 2 viruses or vice versa. In the case of Q461d1, the envelope is highly sensitive to neutralization because of conformational changes that expose multiple epitopes [29]. However, we have found that Q461d1 is relatively insensitive to neutralization by the PGT-type antibodies that recognize a quaternary structure that includes an Asn at amino acid position 160 (unpublished). SF162 is also not recognized by PG9 and certain PGT antibodies because it encodes a Lys at position 160 [4], [8]. Interestingly, the virus that initially infected QA413 encodes an Asn at position 160, while the superinfecting virus encodes a Lys, perhaps suggesting that PGT-like antibodies could contribute to the antibody response in this woman. Mapping studies to define the epitope specificity will be needed to understand the unusual pattern of virus neutralization observed in QA413 pre- and post-SI.

In this study, we examined breadth and potency after SI against a panel of heterologous viruses. In a prior study, the responses to autologous viruses were examined in five of these individuals [13]. Interestingly, one of the elite neutralizers, QA013, developed very high titers against her superinfecting virus, with IC50 values >1,800 (range: 1,000–25,000+) to all four SI variants cloned at ∼5 years post-SI (6.3 years post-initial infection). The other four superinfected individuals, who did not develop elite responses, had autologous responses that ranged from <100 to 10,000. There was insufficient data to determine if there was an association between autologous and heterologous responses.

It is difficult to directly compare the NAb responses of the superinfected women to broad neutralizers identified in other studies because there is no standard for quantifying NAb breadth against HIV-1. We addressed this issue by using a diverse panel of viruses weighted towards Tier 2 variants and several alternate breadth scoring methods, which showed that no single virus drove the association observed between SI and NAb breadth and that our conclusion is the same irrespective of the scoring method used. Simek et al. previously defined elite activity as “the ability to neutralize, on average, more than one pseudovirus at an IC50 titer of 300 within a clade group and across at least four clade groups [9].” While we were unable to completely satisfy these criteria since our 8-virus panel included only single variants of subtypes B and D, we still found that the two superinfected individuals (QA013 and QB850) with the broadest responses could neutralize at least two viruses in subtypes A and C, as well as both single viruses tested from subtypes B and D at an IC50 titer greater than 300, supporting the characterization of these individuals as elite neutralizers. Notably, plasma antibodies from QB850 neutralized viruses from all four subtypes tested at IC50 values greater than 600, more than 2-fold higher than the bar set for elite neutralizers [9]. Furthermore, the responses of these two women were greater than those found in a similar screen of 70 singly infected women in this cohort at ∼5 years post-initial infection [11], with some observed IC50 titers against Tier 2 viruses 6-fold more potent than those of the top 10% of the singly infected women (data not shown). Although we had limited opportunities to directly compare the responses of the superinfected individuals studied here and individuals identified as elite neutralizers by Simek et al. in the IAVI cohort, we did find that the SI cases had IC50 values that were either comparable or above the cohort GM IC50 for the three viruses tested in common between the studies. Together, these data suggest that 2 of the 12 individuals from our SI cohort developed NAbs that exhibit elite activity. This is a remarkable fraction (17%) of individuals with elite activity, although because of differences between screening methods, it is difficult to compare this to the 1% of presumably singly infected elite neutralizers reported previously by Simek et al.

In a prior study that examined four cases of SI, a greater increase in NAb breadth post-SI among superinfected individuals compared to singly infected individuals was also observed [30]. However, it is hard to compare their data with our own, as the previous study used randomly chosen primary isolates for measuring breadth in a cohort with unknown seroconversion dates. It is also interesting to note that this previous study observed a decrease in viral load in three of the four superinfected cases studied at a time point post-SI, two of which had undetectable viral loads. Such a significant drop in viral load has not been previously reported for cases of SI [31]. In contrast, we observed an increase or no change in viral load in the majority of superinfected women examined. This is consistent with the observation that viral load is highly correlated with NAb breadth [11], [16]. However, after adjusting our breadth score analysis for contemporaneous viral load, the estimate of 1.68 was unchanged, while adjusting for this variable caused an increase in our estimate for differences in potency. This would imply that breadth in these superinfected cases alone cannot be explained entirely by an increase in viral load following SI, but rather suggests that stimulation from antigenically distinct viruses may contribute to the development of potency.

Finally, we found evidence of cross-subtype breadth, including detectable neutralization of viruses from four different subtypes in the two women with elite NAb responses within 1 year of their SI. Because both of these individuals were superinfected soon after their initial infection, these cross-subtype responses arose relatively soon after HIV-1 seroconversion. Indeed, by 2 years after their initial infection, both women had antibodies capable of neutralizing 7 of the 8 primarily Tier 2 viruses tested. Recent studies suggest that cross subtype breadth is rare before 2 years post-infection in individuals who are presumably singly infected [17], [32]. Our findings raise the interesting possibility that some of the individuals identified as having broad responses in prior screens may have been superinfected.

A few caveats to these findings must be considered. First, there may have been potential for misclassification of singly infected women, due to our limit of detection or if recombination occurred between the initial and superinfecting strains in HIV-1 genomic regions outside of *gag* and *env* [21]. However, this misclassification would be expected to decrease our ability to detect differences between superinfected and singly infected women, making it more likely that the true association between SI and NAb breadth is stronger than what we observed. Also, we cannot exclude the possibility that there are other factors involved with the development of the broad responses in some of the superinfected women.

This study reveals an unexplored source of naturally-occurring broadly NAbs, and represents a highly relevant approach to inform vaccine strategies in three key ways. First, studies on this and other SI cohorts may provide additional support for immunizing with particular combinations of different HIV-1 strains could be an effective vaccine approach [33]–[37]. Given that the greatest breadth was observed in cases of intersubtype SI, the use of Env immunogens from different subtypes may be optimal. Second, the NAbs and viruses isolated from members of this cohort may hold important clues to antigenic determinants capable of eliciting cross-reactive antibodies that can protect against multiple subtypes of HIV-1. Third, longitudinal studies of superinfected individuals who develop broad and potent NAb responses, such as those identified here, may foster an understanding of the mechanism leading to the elicitation of breadth. Of particular interest is the observation that two elite neutralizers developed broad and potent responses soon after infection by a second HIV-1 strain, suggesting that the processes that lead to the development of broad HIV-specific NAbs are accelerated by a second infection. If SI ultimately leads to the rapid capacity of the overall NAb response to recognize diverse circulating HIV-1 variants, a successful vaccination strategy that mimics natural SI may lead to the development of broad NAb in immunized individuals.

## Materials and Methods

### Ethic statement

The University of Washington's, University of Nairobi and Fred Hutchinson Cancer Research Center's Institutional Review Boards approved the study. Written informed consent was provided by all study participants.

### Study population and design

The individuals in this study represent a subset of a prospective cohort of HIV-1 negative high-risk women from Mombasa, Kenya [38]–[40] who have a defined date of infection based on approximately monthly HIV-1 serology and subsequent retrospective RNA testing of banked plasma from time points prior to seroconversion as described [41]. Twelve superinfected individuals were identified from a previous screen of 56 women from this cohort that compared partial *env* and/or *gag* sequences amplified from peripheral blood mononuclear cells (PBMCs) from the first visit after the detection of seroconversion and a visit during chronic infection ∼5 years later [20]–[22]. Briefly, single copy PCR was performed and the sequences from multiple independent PCRs (median of 7) were examined at each time point. Two regions of the HIV-1 genome were examined: envelope V1-V5 (∼1.2 kb) and gag p17 (∼700 bp). Individuals identified as potential SI cases were further analyzed to verify SI and determine the interval when it occurred. All cases of SI as well as the approximate timing of SI were determined by phylogenetic analysis and allele-specific PCR tailored to the sequences in each individual. For this study, three singly infected women from the 56 tested for SI in the prior study [20]–[22], were matched to each of the 12 cases of SI according to initial infecting viral subtype and sample availability at time points approximately 5 years post-initial infection (post-SI) and 1 year prior to SI (pre-SI). In cases where samples close to 5 years post-initial infection were not available for the SI case, the closest available sample post-SI was selected and the timing of sample selection was similar for the controls (Figure 2). Other than sample availability, the assignment of controls was random, and was performed using random number generation. Viral loads were determined by Gen-Probe and were available for all women, and CD4^+^ T cell counts were documented beginning in 1998 and were available for 17 women prior to SI and 45 women post SI [42]–[44]. All women were HIV-1 infected through heterosexual contact [38], and none reported using antiretroviral therapy during follow-up for this study.

### Heterologous pseudovirus panel

The panel of eight viruses was chosen to include multiple subtypes with varying neutralization sensitivities to a pool of HIV+ plasma from the Mombasa cohort and monoclonal antibodies (i.e. b12, 4E10 [45], [46]). The viruses used, and their subtypes in parenthesis, were: SF162 (B) [47], Q461d1 (A) [45], Q842.d16 (A) [45], QD435.100M.a4 (D) [45], DU156.12 (C) [48], QC406.70M.f3 (C) [46], Q259.d2.26 (A) [45], Q769.b9 (A) [45]. Plasma was also tested against an envelope from Simian Immunodeficiency Virus (SIV), SIVmne CL8 [49], to ensure that the neutralization observed against the virus panel was HIV-1-specific. The two elite neutralizers identified in the study were also tested against JR-CSF (B) [50], 6535.3 (B) [51], CAAN5342.A2 (B) [51], and QB857.23I.B3 (D) [46]. Pseudoviruses were made by cotransfecting 293 T cells with each of the cloned viral envelopes listed above and a full-length subtype A proviral clone with a partial deletion in envelope (Q23Δenv), as previously described [52]. Briefly, an equimolar ratio of envelope-to-provirus plasmid was added to Fugene-6 transfection reagent (Roche, Indianapolis, IN) and then incubated with 4 million 293 T cells for 12 hours. The media was changed at 10 hours. After a total of ∼48 hours post-transfection, supernatants were harvested and filtered through a 0.22 um Steriflip Filter Unit (Millipore, Billerica, MA) to remove cellular debris. The resulting pseudoviruses were screened for infectivity on TZM-bl cells, a HeLa-derived reporter cell line that expresses high levels of CD4, CCR5, and CXCR4 as well as B-galactosidase under the transcriptional control of HIV-LTR [53]. Infectious titers were determined by serially diluting viruses 10-fold, adding 20,000 TZM-bl cells in growth media containing 20 ug/mL DEAE-dextran per well, and incubating at 37°C for 48 hours. The cells were then fixed and stained for B-galactosidase activity and infected cell foci were enumerated visually.

### Neutralization assay

The TZM-bl neutralization assay was used to quantify NAb breadth as previously described [45]. Briefly, 500 infectious pseudovirus particles, as determined by the infectious titer described above, were incubated in duplicates with 2-fold serial dilutions of plasma for 1 hour, beginning with an initial concentration of 1∶100, before 10,000 TZM-bl reporter cells per well were added. Each plasma-virus combination was tested in duplicate and the assay was repeated twice. Infection levels were determined by B-galactosidase activity after 48 hours using a chemiluminescent readout. The IC50, or reciprocal plasma dilution at which 50% of the virus is neutralized, for each plasma-virus pair was calculated using linear interpolation from the neutralization curve. In this assay, a plasma sample was considered to be below the detectable limit of neutralization for a given virus if the lowest dilution (1∶100) did not show >50% neutralization. With this criterion, plasma samples that showed neutralization below the limit of detection were designated an IC50 value of 50, the midpoint between our starting dilution (1∶100) and 0. Each round of assays included HIV-negative plasma and a HIV-positive plasma pool from 30 HIV-1 infected individuals in Kenya between 1998–2000 [45] serving as negative and positive internal controls, respectively. If a run showed neutralization of the negative control virus (SIVmne CL8) greater than the limit of detection, we considered that a failed run and repeated the assay.

### Calculation of breadth and potency scores

Results from two independent experiments were averaged on the log scale before calculating breadth and potency scores. A composite breadth score for each plasma-virus pair was derived for each woman by comparing the IC50 for her plasma to the cohort median IC50 for each virus as described [13]. The cohort median IC50 was based on all IC50s from the individuals in the study, and this represented the unique neutralization sensitivity of that virus. If the IC50s for the given plasma-virus pair was greater than the cohort median IC50, then individuals were given a score of 1, while those below were scored as a 0. The overall breadth was then a composite score for each individual against all eight viruses in the panel, with a maximum score of 8 and a minimum of 0. We also analyzed our dataset using the percent neutralization at the first three dilutions in our assay (1∶100, 1∶200, 1∶400) and applied the same breadth scoring method. Potency was calculated by dividing the IC50 value for a given plasma-virus combination by the cohort median IC50 for that virus. The overall potency was then a composite score for each individual against all eight viruses in the panel.

A method described in Simek et al, was also applied to the data. In this method, for each virus, the average log-transformed titer is calculated for a given sample then scaled to yield a value between 0.0 and 1.0 [9]. As a minor modification to take into account the varying sensitivities in our virus panel, we divided the average by the maximum value for the cohort. An average of the scaled averages across viruses was computed as a final breadth score for each individual.

### Statistical analysis

Breadth scores were analyzed using conditional Poisson regression, while log transformed potency scores were analyzed using linear regression generalized estimating equation (GEE). These models were appropriate given the observed mean-variance relationships of the breadth and potency scores. The breadth scoring method described by Simek et al. was analyzed using GEE. All models assessed by GEE used an identity link, exchangeable working correlation structure, and robust standard errors. We used a paired t-test to compare viral load, CD4+ T cell count and geometric mean IC50s between superinfected and non-superinfected individuals. Spearman's rank correlation was used for comparing the results from our final model and single dilution analysis. Two-tailed P values of 0.05 or less were considered to indicate significance in all statistical tests. Analyses were performed using STATA statistical software (version 11, StataCorp, College Station, TX).

## Supporting Information

Table S1 Sensitivity analysis to assess whether the association between SI and NAb breadth was variable depending on the breadth scoring method or viruses used to test for neutralization activity. Breadth scores were derived using methods from Simek et al. and Blish et al. and then compared between superinfected cases and non-superinfected controls using GEE and Poisson Regression, respectively. These analyses were further subjected to examination with a stepwise removal of neutralization data from one virus at a time and inclusion or exclusion of various combinations of viruses from the original 8-virus panel. The point estimates for each scenario described are listed in separate sections for both Simek and Blish scoring methods.
(PDF)

Table S2 Determining the relationship between SI and NAb breadth using a breadth scoring method based on the percent neutralization from a single dilution of plasma (1∶100, 1∶200, or 1∶400). The original RR assessed with breadth scores derived from IC50s using serial dilutions is listed first for comparison, while the RRs assessed with breadth scores derived from percent neutralization at a single dilution are listed below.
(PDF)

Table S3 Spearman's rank correlation between breadth scores derived from IC50s using serial dilutions versus scores using percent neutralization at a single dilution.
(PDF)
