# Supplementary material for: HIV-1 Superinfection in Women Broadens and Strengthens the Neutralizing Antibody Response
Source: PLoS Pathog. 2012 Mar 29;8(3):e1002611. doi: 10.1371/journal.ppat.1002611 (PMC3315492; doi:10.1371/journal.ppat.1002611)
Supplement: Table S1 — Sensitivity analysis to assess whether the association between SI and NAb breadth was variable depending on the breadth scoring method or viruses used to test for neutralization activity. Breadth scores were derived using methods from Simek et al. and Blish et al. and then compared between superinfected cases and non-superinfected controls using GEE and Poisson Regression, respectively. These analyses were further subjected to examination with a stepwise removal of neutralization data from one virus at a time and inclusion or exclusion of various combinations of viruses from the original 8-virus panel. The point estimates for each scenario described are listed in separate sections for both Simek and Blish scoring methods. (PDF) [file ppat.1002611.s001.pdf]

		Coefficient	95% CI	P value
**Simek scoring [9] GEE**	**8-virus panel**	0.18	(0.07-0.30)	0.002
	**Tier 2 viruses***	0.23	(0.09-0.37)	0.002
	**Most resistant viruses^**	0.29	(0.13-0.45)	<0.0005
	**Medium sensitivity#**	0.18	(0.06-0.30)	0.003
	**Only subtype A**	0.20	(0.06-0.34)	0.005
	**Non-subtype A**	0.16	(0.07-0.26)	0.001
	**Sans SF162**	0.20	(0.07-0.33)	0.002
	**Sans Q461d1**	0.20	(0.08-0.33)	0.002
	**Sans Q769.b9**	0.16	(0.04-0.27)	0.006
	**Sans Q842.d16**	0.19	(0.08-0.31)	0.001
	**Sans Q259.d2.26**	0.17	(0.07-0.27)	0.001
	**Sans Q435.100M.a4**	0.18	(0.05-0.30)	0.007
	**Sans Q406.70M.f3**	0.18	(0.07-0.28)	0.001
	**Sans DU156.12**	0.19	(0.07-0.31)	0.002

		RR	95% CI	P value
**Blish scoring [13] Poisson regression**	**8-virus panel**	1.68	(1.25-2.26)	0.001
	**Tier 2 viruses***	1.80	(1.28-2.52)	0.001
	**Most resistant viruses^**	1.91	(1.19-3.07)	0.007
	**Medium sensitivity#**	1.80	(1.29-2.51)	0.001
	**Only subtype A**	1.65	(1.20-2.26)	0.002
	**Non-subtype A**	1.72	(1.26-2.35)	0.001
	**Sans SF162**	1.75	(1.28-2.40)	<0.0005
	**Sans Q461d1**	1.71	(1.25-2.34)	0.001
	**Sans Q769.b9**	1.62	(1.18-2.23)	0.003
	**Sans Q842.d16**	1.36	(1.30-1.42)	<0.0005
	**Sans Q259.d2.26**	1.37	(1.31-1.42)	<0.0005
	**Sans Q435.100M.a4**	1.62	(1.18-2.23)	0.003
	**Sans Q406.70M.f3**	1.38	(1.32-1.45)	<0.0005
	**Sans DU156.12**	1.71	(1.25-2.34)	0.001

*Excluding SF162 and Q461d1
^Q769.b9, Q259.d2.d26, Q435.100M.a4
#Q842.d16, Q435.100M.a4, Q406.70M.f3, DU156.12
