# Supplementary material for: HIV-1 Superinfection in Women Broadens and Strengthens the Neutralizing Antibody Response
Source: PLoS Pathog. 2012 Mar 29;8(3):e1002611. doi: 10.1371/journal.ppat.1002611 (PMC3315492; doi:10.1371/journal.ppat.1002611)
Supplement: Table S2 — Determining the relationship between SI and NAb breadth using a breadth scoring method based on the percent neutralization from a single dilution of plasma (1∶100, 1∶200, or 1∶400). The original RR assessed with breadth scores derived from IC50s using serial dilutions is listed first for comparison, while the RRs assessed with breadth scores derived from percent neutralization at a single dilution are listed below. (PDF) [file ppat.1002611.s002.pdf]

Dilution	RR	95% CI	P value
Serial dilutions	1.68	(1.25-2.26)	0.001
1:100	1.70	(1.30-2.28)	0.001
1:200	1.55	(1.14-2.11)	0.005
1:400	1.58	(1.16-2.13)	0.004
