# Supplementary material for: HIV-1 Superinfection in Women Broadens and Strengthens the Neutralizing Antibody Response
Source: PLoS Pathog. 2012 Mar 29;8(3):e1002611. doi: 10.1371/journal.ppat.1002611 (PMC3315492; doi:10.1371/journal.ppat.1002611)
Supplement: Table S3 — Spearman's rank correlation between breadth scores derived from IC50s using serial dilutions versus scores using percent neutralization at a single dilution. (PDF) [file ppat.1002611.s003.pdf]

Dilution	Rho	P value
1:100	0.85	<0.0005
1:200	0.88	<0.0005
1:400	0.76	<0.0005
